# Two-Step Approach for Occupancy Estimation in Intensive Care Units Based on Bayesian Optimization Techniques

**DOI:** 10.3390/s23031162

**Published:** 2023-01-19

**Authors:** José A. González-Nóvoa, Laura Busto, Silvia Campanioni, José Fariña, Juan J. Rodríguez-Andina, Dolores Vila, César Veiga

**Affiliations:** 1 Galicia Sur Health Research Institute (IIS Galicia Sur), Álvaro Cunqueiro Hospital, 36310 Vigo, Spain; 2 Department of Electronic Technology, University of Vigo, 36310 Vigo, Spain; 3 Intensive Care Unit Department, Complexo Hospitalario Universitario de Vigo (SERGAS), Álvaro Cunqueiro Hospital, 36213 Vigo, Spain

**Keywords:** artificial intelligence, automated machine learning, Bayesian optimization, ICU occupancy, intensive care unit, length of stay, machine learning, MIMIC, XGBoost

## Abstract

Due to the high occupational pressure suffered by intensive care units (ICUs), a correct estimation of the patients’ length of stay (LoS) in the ICU is of great interest to predict possible situations of collapse, to help healthcare personnel to select appropriate treatment options and to predict patients’ conditions. There has been a high amount of data collected by biomedical sensors during the continuous monitoring process of patients in the ICU, so the use of artificial intelligence techniques in automatic LoS estimation would improve patients’ care and facilitate the work of healthcare personnel. In this work, a novel methodology to estimate the LoS using data of the first 24 h in the ICU is presented. To achieve this, XGBoost, one of the most popular and efficient state-of-the-art algorithms, is used as an estimator model, and its performance is optimized both from computational and precision viewpoints using Bayesian techniques. For this optimization, a novel two-step approach is presented. The methodology was carefully designed to execute codes on a high-performance computing system based on graphics processing units, which considerably reduces the execution time. The algorithm scalability is analyzed. With the proposed methodology, the best set of XGBoost hyperparameters are identified, estimating LoS with a MAE of 2.529 days, improving the results reported in the current state of the art and probing the validity and utility of the proposed approach.

## 1. Introduction

The intensive care units (ICUs) of hospitals have a variety of devices to monitor the patients’ health states that generate a large amount of data, allowing healthcare personnel to be aware of the patient’s vital sign values and to make the most appropriate decisions to ensure their correct evolution [1]. It is very difficult to analyze all of this volume of information manually, so it is of great interest to use artificial intelligence (AI) tools that automate and help in these tasks [2], especially in extraordinary situations such as the one experienced due to the COVID-19 pandemic, where ICUs were overwhelmed.

The patient’s ICU length of stay (LoS) is an important metric from a clinical point of view. Due to the high occupational pressure suffered by the ICU, a correct estimation of the patients’ LoS in the ICU is of great interest to predict possible situations of collapse, to help healthcare personnel to select appropriate treatment options and to predict patients’ conditions. The design of a reliable estimator system is useful to anticipate future collapse situations and to take the corresponding actions [2], such as conditioning an area similar to the ICU urgently that accommodates future patients. In this field there are several studies. The vast majority of them approach the problem as a binary classification, predicting, for example, which patients will stay in the ICU for more or less than a defined number of days [3,4,5]. However, other studies approach the problem from the point of view of the exact calculation of LoS [6,7,8], which allows for a finer prediction of the ICU situation. In this article, it was decided to approach the problem from this second point of view, estimating the ICU stay duration from the monitoring data obtained during the first 24 h since the moment the patient accessed the ICU. In order to obtain more precise results, it was decided to delve into the optimization of the hyperparameters of the model, a task which requires a high computational load.

One of the goals of high-performance computing systems is to reduce the execution time of a given task [9]. Due to the increased computational load of artificial intelligence problems, it is necessary to use these systems to reduce the execution time. In recent years, performance enhancement from one processor generation to another has stagnated, making it necessary to find other ways to optimize the performance of algorithms. One of the main alternatives is the use of GPUs (graphics processing units), which help to continue to reduce execution times.

Occupancy prediction is a frequent topic within the healthcare ecosystem [10,11], as well as in other related fields, such as building energy systems [12], building performance [13] and heating, ventilation and air conditioning (HVAC) system control [14]. In this work, we propose a new methodology based on a novel two-step Bayesian optimization approach to improve LoS estimation methods used to predict ICU occupancy. This method allows one to optimize an LoS XGBoost predictor that uses clinical variables extracted from ICU monitoring, by finding the best set of hyperparameters of the model. The proposed approach improves the results of LoS predictions that would be obtained in the case of carrying out the optimization in the conventional way (regular Bayesian) and also improves the results obtained in other state-of-the-art works [3,4,5,6,7,8].

Due to the high computational load that this task entails, an important part of the methodology is the parallelization of the problem on a GPU architecture, allowing it to be solved in a computationally efficient way. XGBoost is used as estimator model. All these contributions are supported by experimental results.

The remainder of the article is structured as follows. First, the materials used are detailed. Then, the proposed methodology is explained and the experimental results are presented. Finally, the discussion and conclusions of the work are presented.

## 2. Materials

### 2.1. Data Source

In this work, the MIMIC-III (Medical Information Mart for Intensive Care III) [15] ICU database, developed by the MIT (Massachusetts Institute of Technology), was used. It contains data from 61,532 ICU stays at Beth Israel Deaconess Medical Center.

**Figure 1:**
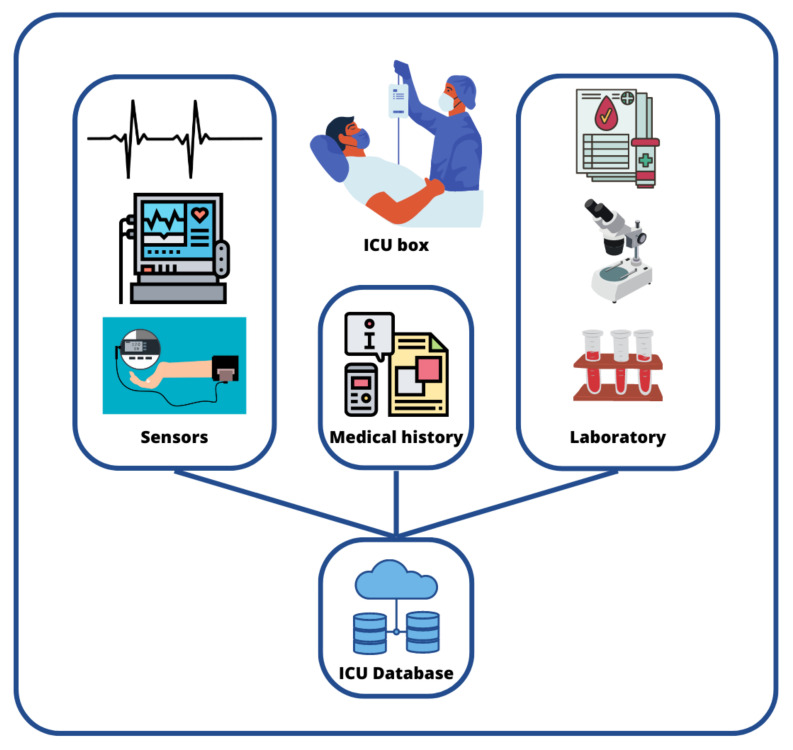
Intensive care unit structure.

The database collected demographic data, vital sign measurements made at the bedside (1 data point per hour), laboratory test data, procedures, medications, caregiver notes, imaging reports, signals (electrocardiography (ECG), photoplethysmogram (PPG), arterial blood pressure (ABP)), etc. It contains the variable to be estimated in this work (LoS in the ICU) with a resolution of ±$10^{-4}$ days (±8.64 s). Figure 1 shows the different data sources of an ICU. The detailed structure of the database can be found in the MIMIC-III original publication [15].

### 2.2. XGBoost

XGBoost [16] is a gradient boosting technique based on ensemble learning. These techniques correct errors made by previous models in successive ones, optimizing a loss function. This function (1) is modified at each iteration *t*. The successive models are built using the exact greedy algorithm, which analyzes all possible split loss reduction options, until the stop condition is achieved. A more detailed explanation may be found in [16].

(1) $$L^{\left(t\right)}=\sum_{i=1}^{n}l\left(y_{i},{\hat{y}}_{i}^{\left(t-1\right)}\right)+\Omega \left(f_{t}\right)$$

where *l* is a differentiable convex loss function that must be transformed into another one in a Euclidean domain using Taylor’s Theorem, the pair $\left(y_{i},x_{i}\right)$ represents the training set, ${\hat{y}}_{i}$ is the final prediction and $\Omega (f_{t})$ is the regularization term used to penalize more complex models through both Lasso and Ridge regularization and to prevent overfitting.

XGBoost is one of the most popular algorithms in the state of the art [17], highlighting its computational efficiency and GPU support. It is one of the most used algorithms in recent biomedical works based on tabular data [18,19,20].

As stated above [21,22], XGBoost uses three types of parameters: general, booster and learning task parameters. General parameters indicate the booster used: tree or linear model. Booster parameters are related with the booster employed and define the internal performance parameters, e.g., learning rate or number of estimators, while learning task parameters indicate the corresponding learning objective. The methodology presented in this work focuses on learning task parameters seeking to optimize the performance of the XGBoost regressor model. These parameters are:

- Learning rate: Step-size shrinkage used in updates to prevent overfitting. After each boost step, the weights of the new features can be obtained directly, and the learning rate reduces the weights of the features to make the boost process more conservative.
- Maximum delta step: the maximum delta step each leaf output is allowed to be.
- Maximum depth of a tree: increasing this value will make the model more complex.
- Maximum leaves: maximum number of nodes to be added.
- Minimum child weight: Minimum sum of instance weight (hessian) needed in a child. If the tree partition step results in a leaf node with the sum of instance weight being less than the minimum child weight, then the building process will give up further partitioning.
- Number of estimators: The number of trees in the ensemble. It is equivalent to the number of boosting rounds.
- Region alpha: L1 regularization term on weights. Increasing this value will make the model more conservative.
- Region lambda: L2 regularization term on weights. Increasing this value will make the model more conservative. It is normalized to number of training examples.
- Scale pos weight: controls the balance of positive and negative weights, useful for unbalanced classes.
- Subsample: Subsample ratio of the training instances. Setting it to 0.5 means that XGBoost would randomly sample half of the training data prior to growing trees. Subsampling will occur once in every boosting iteration.

### 2.3. Bayesian Optimization

One of the relevant parts of a machine learning pipeline is the optimization of the estimator model. This task implies a high computational load, so optimizing it efficiently is a key factor. Given a dataset *D*, the goal of hyperparameter optimization [23] is to find λ in (2):

(2) $$\lambda^{*}=\arg\min_{\lambda \epsilon \Lambda }{I\;E}_{D_{train},D_{test}\epsilon D}V\left(L,A_{\lambda },D_{train},D_{test}\right)$$

where λ is a vector of hyperparameters from the hyperparameter search space Λ, A is the predictive model and $V(L,A_{\lambda },D_{train},D_{test})$ measures the loss of the model A.

In this work, Bayesian techniques [24] were used to carry out this task, which stand out for their computational efficiency when performing the search. The surrogate model (a Gaussian stochastic probabilistic model) was used. The optimization was divided into several stages: The first stage was the set-up of the surrogate model. Next, the best hyperparameter combination was sought, and it was applied to the real objective function. Finally, the surrogate model was updated. This process was repeated iteratively until the defined criteria were achieved.

One of the most used objective functions is that of expected improvements (3), because it can be calculated in closed form if the estimator model *y* with the configuration λ follows a normal distribution (4) [23].

(3) $$I\;E\left[I\;I\left(\lambda \right)\right]=I\;E\left[max\left(f_{min}-y,0\right)\right]$$

(4) $$I\;E\left[I\;I\left(\lambda \right)\right]=\left(f_{min}-\mu \left(\lambda \right)\right)\Phi \left(\frac{f_{min}-\mu \left(\lambda \right)}{\sigma }\right)+\sigma \phi \left(\frac{f_{min}-\mu \left(\lambda \right)}{\sigma }\right)$$

where ϕ(·) and Φ(·) are the standard normal density and standard normal distribution, and $f_{min}$ is the best observed value.

In this work, the open-source package Hyperopt [25], which uses Bayesian optimization as the search technique, was used.

## 3. Methodology

**Figure 2:**
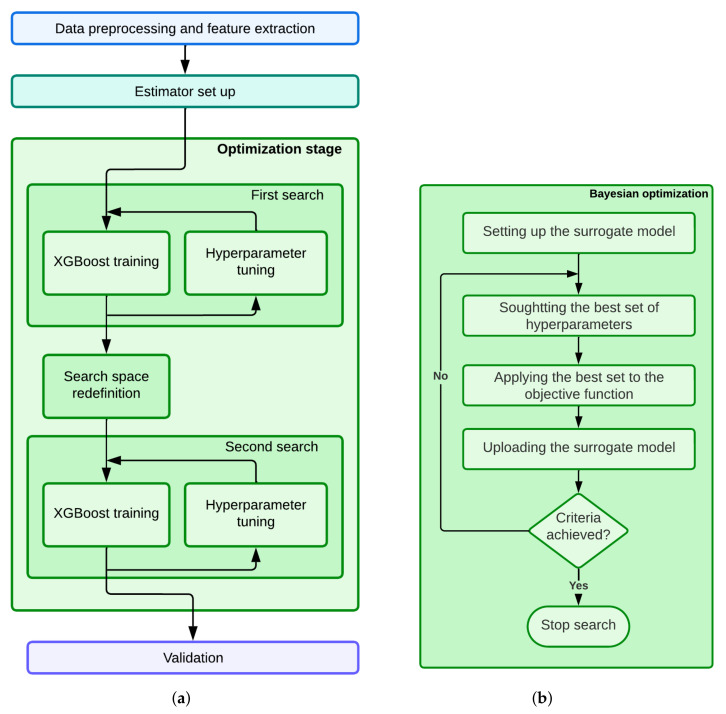
Outline of the proposed methodology. (**a**) Shows a general outline, while a more detailed explanation of the hyperparameter search using Bayesian optimization is shown in (**b**).

The proposed methodology to optimize the estimator model of the patient’s LoS is divided into several stages. Figure 2a shows an outline of these stages, while a more detailed explanation of the hyperparameter search using Bayesian optimization is shown in Figure 2b. The first stage is related to the data preprocessing and the feature extraction. The second one is devoted to building the LoS estimator. In this work, we propose to use the state-of-the-art approach based on gradient boosting, namely XGBoost, due to the good results obtained in estimation tasks and the high level of computational improvement that could be achieved on a GPU architecture during the optimization stage. The third stage is devoted to optimizing the model hyperparameters in order to improve the performance of the estimator. In this work, a novel two-step Bayesian optimization approach is proposed, implemented on a GPU architecture to reduce the execution time. Finally, the estimator model is validated.

### 3.1. Data Preprocessing and Feature Extraction

The first stage of the pipeline was devoted to data preprocessing to obtain a set of features, derived from the clinical data, used to fit and validate the estimator model. Such a stage required two tasks.

The first task consisted of selecting the variables used to produce the dataset from the original database. In this approach, all available clinical variables were considered, selecting only the ones that were present in at least 80% of the patients for building the model, as in other published works [21,26]. The reason why all of the variables were not available for all patients was because, depending on the pathology and the patient’s clinical condition, only a certain set of variables was monitored. Patients who did not have values in at least 2/3 of these variables were subsequently discarded. In order to deal with missing data, a schema for input data was required to fill values on such empty variables. Although there are other imputation methods, such as MissForest [27] or generative adversial networks [28], in this work we have proposed using K–Nearest Neighbors, as it is one of the most widely used in the current state of the art, in addition to its simplicity in implementation and usage.

The next task consisted of the conversion from variables to features, in this case by computing the mean value, standard deviation and maximum and minimum values of the clinical variables gathered during the first 24 h of the patient’s ICU stay, except for the volume of urine, for which only the total volume in this time interval was used.

### 3.2. LoS Estimation Model Building

Once the previous preprocessing stage was completed, it was necessary to proceed with the configuration stage of the ICU LoS estimator model. As mentioned above, the XGBoost model was used, which was fitted using the above described features. In order to train the model, a random split train and test of these data was carried out following a ratio of 80/20. To validate the model, the mean absolute error (MAE) (5) was used, which is one of the most popular metrics in the current state of the art, allowing for the comparison of the results.

(5) $$MAE=\sum_{i=1}^{n}\left|{\hat{y}}_{i}-y_{i}\right|$$

where *n* is the number of patients, ${\hat{y}}_{i}$ is the LoS estimated by XGBoost model and $y_{i}$ is the golden standard for the LoS, obtained from the database.

### 3.3. XGBoost Optimization: Two-Step Approach

To improve the accuracy of the model, we delved into the automatic optimization of the hyperparameters of XGBoost instead of using the default values.

This stage was divided into two steps. A first search step was executed using the initially defined hyperparameter search space. Then, a second search was performed once the search space was modified after the hyperparameters’ evolution during the first search stage was analyzed.

The optimization started with the hyperparameter search space (Λ) definition, which consisted of indicating which parameters should be varied during the different iterations (2) and within which limits, in addition to indicating the type of search space. There are three types of search space distributions: uniform, log uniform and q uniform, which return real values uniformly distributed between defined limits. Log uniform is more suitable for geometric series, whereas uniform and q uniform are more suitable for arithmetic series, with the difference being that q uniform returns round values, so the selection of the search space depends on the hyperparameter type. This part was fundamental, since an incorrect definition of space can cause the process to not work efficiently in computational terms.

In addition to defining the search space, it was necessary to define an objective function to minimize in each iteration, that is, the statistical metric that defined the quality of the estimator model needed to be defined. In this work, it was decided to use the MAE (5) as a metric. Firstly, the maximum number of evaluations (*m*) was defined as 5000.

Once this first search was completed, the search space was modified to improve the results obtained avoiding the bottlenecks derived from the initial defined limits and refocusing on the area where the best value was found after the first search, but expanding the search limits, as shown in the Results section. Table 1 summarizes the search space distribution used in the first and second phases, and the types of distributions used.

## 4. Results

In this section, the results obtained using the proposed methodology are presented and analyzed, in terms of the set of hyperparameters identified during the optimization stage, the performance of the model estimator using MAE as the metric and the computational performance. For this analysis, the initial dataset is randomly split into train (27,077 patients) and test (6770 patients) sets, calculating the MAE for the test set.

### 4.1. Hyperparameter Tuning Stage

Regarding the hyperparameter optimization stage, the method permits one to reduce the MAE, and consequently improve the estimations of the model, by identifying the set of parameters that provides the lower MAE. Table 2 shows the best combination of hyperparameters obtained both after the first search and after the second search, explained and described in Section 3.

**Figure 3:**
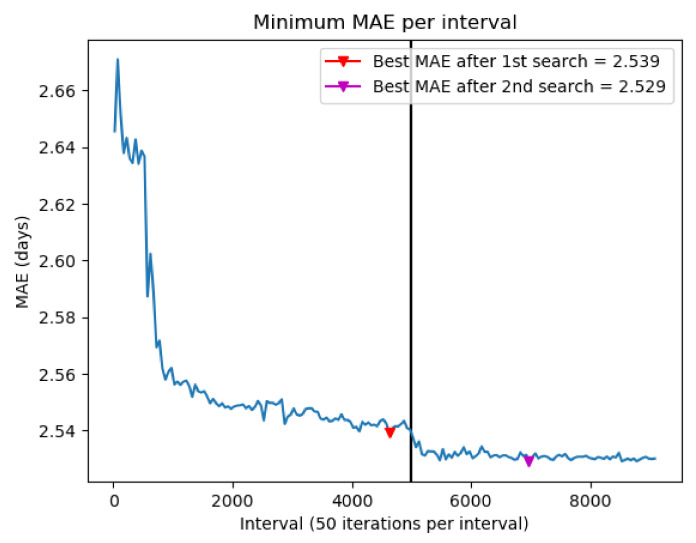
Evolution of minimum MAE across 50 iteration intervals.

The whole evolution of interactions of both optimization steps are plotted in Figure 3, which shows the minimum value of the MAE obtained (grouped into intervals of 50 iterations), that clearly shows the downward trend. The figure also shows how after redefining the search space from the results of the first search step (black vertical line), there is a sharp drop in the value of the MAE, obtaining a minimum value of 2.529 in iteration 6962.

**Figure 4:**
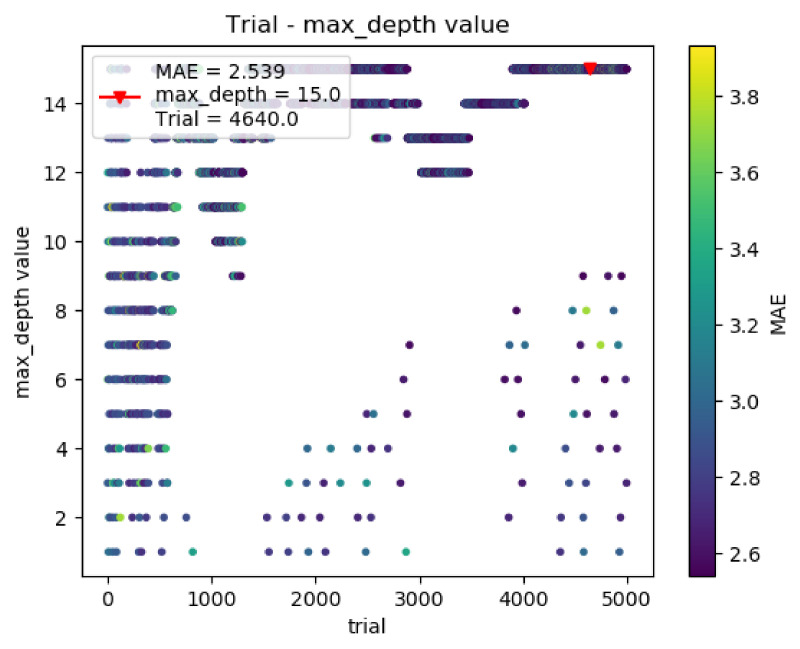
Evolution of maximum depth value and its corresponding MAE across the trials.

Another important element when analyzing the optimization stage of the model is the evolution in the search, that is, how the values of the hyperparameters vary throughout the different trials. Figure 4 shows the value of one of the model hyperparameters (the maximum depth) in each trial, with its corresponding MAE. The figures corresponding to the rest of the hyperparameters are shown in the Supplementary Materials. From these figures, what was indicated above can also be seen, meaning that there is no direct correlation between the value of the individual hyperparameter and the value of the MAE obtained. However, it shows that the intervals between the optimal MAE value obtained and the trend in the search are found. From these results, a second optimization step was performed by redefining the search space of the Bayesian model, modifying the search space limits. After this second optimization step, a MAE of 2.529 was obtained.

### 4.2. Estimator Validation

Only the first stay of each patient was selected in the database to avoid possible information leakage and to compare the results obtained with related works [8], resulting in a total of 46,476 stays. As indicated in Section 3, a second filtering was subsequently carried out to discard patients for whom more that 1/3 of the variables were unavailable, obtaining a total of 33,847 ICU stays. Table 3 shows the main characteristics of the resulting cohort.

Table 4 shows the clinical ICU variables and features extracted from each patient. It consists of a total of 35 variables as 139 features.

To quantify the precision of the model when estimating the patient’s LoS in the ICU, the MAE was used as the metric. As mentioned above, the initial dataset was split into train (27,077 patients) and test (6770 patients) datasets, calculating the MAE for the test set. The LoS was estimated with a resolution of ±$10^{-4}$ days, obtaining a MAE of 2.529 days, lower than the rest of the current state-of-the-art works consulted. Rouzbahman et al. [29] obtain a MAE of 5.07 days, while Alghatani et al. [30] obtain a MAE of 2.64 days, both using MIMIC as the database. Moreover, the final MAE obtained is lower than the MAE obtained with the default hyperparameters (3.040) and after the first optimization step (2.539). Although it is true that there are studies with a slightly lower MAE value, they focus on a specific group of patients, instead of addressing the problem from a generic point of view, as we do. Our work improves the results of those who face problems from a generic point of view. Table 5 shows the results obtained.

### 4.3. Computational Performance

Model fitting and optimization are extremely expensive processes. To carry out this work, a high-performance computing system was required. This architecture consisted of a 24-core CPU, 256 GB of RAM and one NVIDIA A100 GPU unit. The GPU had 40 GB of memory and consisted of 108 multiprocessors, allowing the execution of 2024 threads per multiprocessor, a total of 221,184 threads per GPU [31]. One of the main objectives of the methodology proposed in this article was computational efficiency. For this, it was necessary that the software used could be executed on the GPU.

In order to benchmark the effects that hardware and dataset dimensions have on the performance of the proposed method, several tests were conducted, both on the GPU and on the CPU (using one core and three cores). Each hardware configuration was tested with three different dataset versions, namely the original dataset used in a previous experiment (dataset), this dataset reduced to half of the number of patients (Subdataset 1) and a dataset with half of the features (Subdataset 2). Both the selection of patients and characteristics were performed randomly. Table 6 shows the results obtained in minutes using 100 iterations (*m*).

Once the drastic difference in time between the CPU and GPU was verified, the number of iterations was increased to 500, only using the GPU to analyze how it influenced the execution time. It was observed that the execution time increases practically in the same proportion as the number of iterations. The variation in this proportion is due to the fact that depending on the value of the hyperparameters in which it is iterating, the execution time differs.

## 5. Discussion

The proposed methodology allows one to identify the set of hyperparameters that provides the best performance of the predictor in terms of minimizing the mean absolute error (MAE). With the best combination of hyperparameters, the LoS was estimated with a MAE of 2.529 days, lower than the rest of the current state-of-the-art works consulted. It is also lower than the MAE obtained after the first search stage (MAE = 2.539 days) and lower than using the default XGBoost hyperparameters set without executing the optimization task (MAE = 3.04 days). Although it is true that there are studies with a slightly lower MAE value, they focus on a specific group of patients, instead of addressing the problem from a generic point of view, as was indicated in Section 1.

XGBoost was used as an estimator model due to being one of the ones that obtains the best results in other current state-of-the-art works and its computational efficiency. However, the same methodology could be applied to other models (Random Forest, Support Vector Machine, etc.). The feature extraction stage could be modified adding data from other ICU sensors, extracting other statistics or considering another time window instead of 24 h.

One of the fundamental reasons for obtaining better results is the optimization phase of the model. Adapting the search methodology to work in the GPU allowed us to intensify the search for the best combination of hyperparameters. In addition, as already indicated above, the search technique used based on Bayesian optimization was characterized by performing the search for hyperparameters more efficiently from a computational point of view. The results also confirm that the two-step approach proposed for the optimization of the estimator model improves the results obtained. This same proposal could be applied to another problem.

A stagnation of the MAE improvement is observed with the passing of the trials, which gives rise to an analysis of the relationship of computational cost–improvement obtained, in which it would be necessary to assess the available hardware and the allowable error in each particular situation. Regarding the relationship between the individual values of the hyperparameters and the value of the MAE, no clear patterns are observed, which justifies that what is really relevant is the combination of the values of the different hyperparameters, not the values of each one separately. This demonstrates the need to perform the optimization of the model automatically, as was the case in this work, instead of performing it manually, which would be unfeasible in terms of work times, and worse results would be obtained.

From the computational results, how the use of the GPU drastically reduces the execution time is observed. It is also interesting to analyze how the variations in the size of the dataset hardly imply variations in execution times in the GPU. This makes sense, since although the size of the dataset is reduced, GPUs are characterized by processing large amounts of data in parallel in a single clock cycle, so the variation in the size of the dataset does not always imply a variation in the timing of execution. If the same methodology was used in the classic architectures based solely on the CPU, the execution time would have been drastically higher, which would limit the ability to analyze the possible improvements to be made to the methodology for a real clinical application.

As for future lines of work, this methodology can be extended in several ways. The first line of research is related to the application domain, regarding using this novel methodology to estimate other time variables that are different to LoS, as well as to estimate other variables aside from time within the ICU, and extending the applicability to other hospital areas and by extension to any other social prediction. Another future line of research could be exploring the configuration domain, namely the modifications in the internal configuration of the methodology (the estimator model used to change the XGBoost predictor model by any other regressor model that could provide a time estimation). Modifications in the optimization stage and in the feature extraction stage are also of great interest, using more data sources or using different feature extraction techniques.

## 6. Conclusions

This article presents a methodology to estimate the LoS in the ICU using data collected by UCI sensors and other sources (laboratory and medical history) during the first 24 h of a patient’s admission, focusing on the optimization stage of the estimator model, both from a computational point of view and at the estimation precision level. To do this, the methodology was adapted so that the training of the model was executed on the GPU and the search for the best combination of hyperparameters was carried out automatically using Bayesian optimization techniques in a novel two-step approach.

The results using 33,847 patients demonstrate the validity of the proposed methodology, obtaining a MAE of 2.529 days, lower than that of other works from the current state of the art consulted. In addition, an improvement in model precision by dividing the model optimization phase into two steps instead of performing it in a single step is also demonstrated.

This work opens several future lines of research for applying the present methodology to predict other variables within the ICU, or in other hospital areas. Another future line of research could be exploring modifications in the internal configuration of the methodology (the estimator model used, modifications in the optimization stage, using different features, etc.).

## Figures and Tables

**Table 1:** Hyperparameter distributions.

Hyperparameter	Distribution Type	First Step Distribution		Second Step Distribution	
		Min	Max	Min	Max
Learning rate	loguniform	−8	0	−7.5	−4.5
Max. depth	quniform	1	15	14.5	25
Min. child weight	quniform	0	10	6	12
Max. delta step	quniform	0	10	0	0.5
Subsample ratio	uniform	0.1	1	0.4	0.7
Lambda region	uniform	0.1	1	0.7	1.2
Alpha region	uniform	0.1	1	0.4	0.8
Scale pos weight	uniform	0.1	1	0.45	0.85
Max. number of leaves	quniform	0	10	0.1	1
Number of estimators	quniform	1	10,000	100	2500

**Table 2:** Hyperparameter optimal values.

Hyperparameter	Best Value	
	First Search	Second Search
Learning rate	0.00135	0.00132
Max. depth	15	24
Min. child weight	9	12
Max. delta step	0	0
Subsample ratio	0.525	0.529
Lambda region	0.891	1.184
Alpha region	0.661	0.559
Scale pos weight	0.674	0.755
Max. number of leaves	0	0
Number of estimators	745	812

**Table 3:** Characteristics of the data source.

	MIMIC-III	Resulting Cohort
Number of patients	46,476	33,847
Number of ICU stays	61,532	33,847
Average age	64.93	74.65
Gender	F: 20,380 M: 26,096	F: 19,319 M: 14,528
LoS average	4.92	4.32
LoS standard deviation	9.64	6.21
LoS variance	92.91	38.55

**Table 4:** Features extracted from each clinical variable.

Clinical Variables	Statistics
Age	Value in admission
Gender	F/M
Urine output	Accumulated value after 24 h
Glasgow coma motor scale	
Glasgow coma verbal scale	
Glasgow coma eyes scale	
Systolic blood pressure	
Heart rate	
Body temperature	
PaO_2_	
FiO_2_	
Serum urea nitrogen level	
Sodium level	
Potassium level	
Bilirubin level	
Respiratory rate	
Glucose	
Albumin	
Anion gap	Average, maximum, minimum and standard deviation value after 24 h
Chloride	
Creatinine	
Lactate	
Calcium	
Haematocrit	
Hemoglobin	
INR ^1^	
Platelets	
Prothrombin time test	
Activated thromboplastin time	
Base excess	
PaCO_2_	
FiCO_2_	
PH	
Total CO_2_	

^1^ International normalized ratio of prothrombin time.

**Table 5:** Comparison of the MAE obtained using the default model with respect to that obtained after the first and second steps of the optimization phase.

Default Estimator (Without Optimization)	Optimized Model (After First Step)	Optimized Model (After Second Step)
3.040	2.539	2.529

**Table 6:** Time comparison. *m* is the number of iterations.

	CPU		GPU	
	1 Core (*m* = 100)	3 Core (*m* = 100)	*m* = 100	*m* = 500
Dataset	85.86’	39.26’	9.70’	75.23’
Subdataset 1	82.56’	41.86’	11.82’	63.06’
Subdataset 2	44.63’	24.50’	9.07’	44.63’

## Data Availability

The datasets analyzed in the current study are available in the PhysioNet repository, https://physionet.org/content/mimiciii/1.4/ (accessed on 10 January 2021).

## Supplementary Materials

The following supporting information can be downloaded at: https://www.mdpi.com/article/10.3390/s23031162/s1. Figures similar to Figure 4 are added as Supplementary Material for each hyperparameter.

## Abbreviations

The following abbreviations are used in this manuscript:

CPU	central processing unit
GPU	graphics processing units
ICU	intensive care unit
INR	international normalized ratio
LoS	length of stay
MAE	mean absolute error
MIMIC	Medical Information Mart for Intensive Care
