# Supplementary figures and images for: Two-Step Approach for Occupancy Estimation in Intensive Care Units Based on Bayesian Optimization Techniques
Source: Sensors (Basel). 2023 Jan 19;23(3):1162. doi: 10.3390/s23031162 (PMC9919941; doi:10.3390/s23031162)

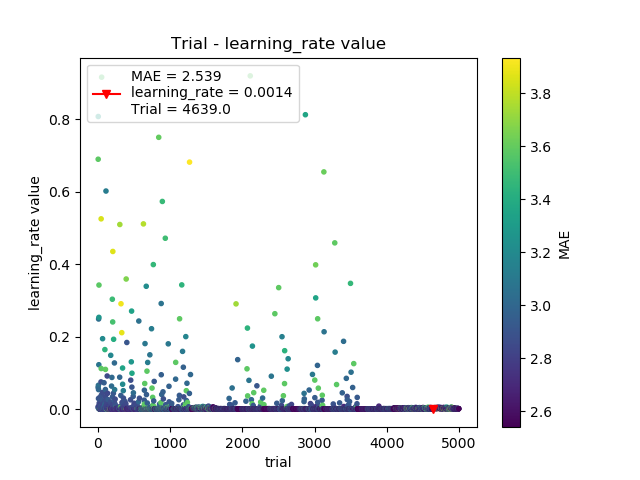

Supplement: Supplementary file 1 [file sensors-23-01162-s001.zip › sensors-2100317-supplementary/FS1.png]

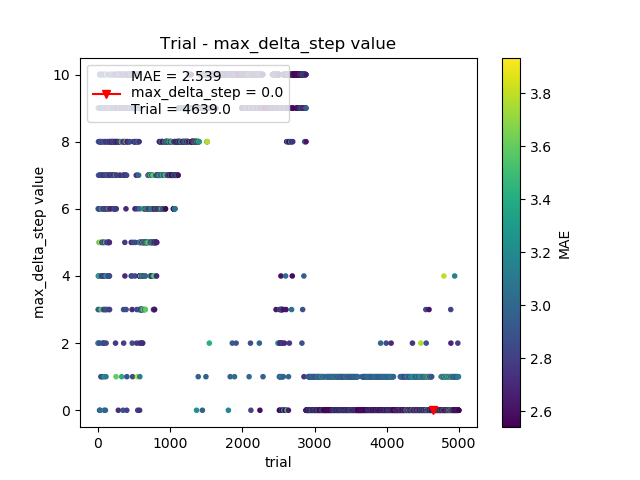

Supplement: Supplementary file 1 [file sensors-23-01162-s001.zip › sensors-2100317-supplementary/FS2.png]

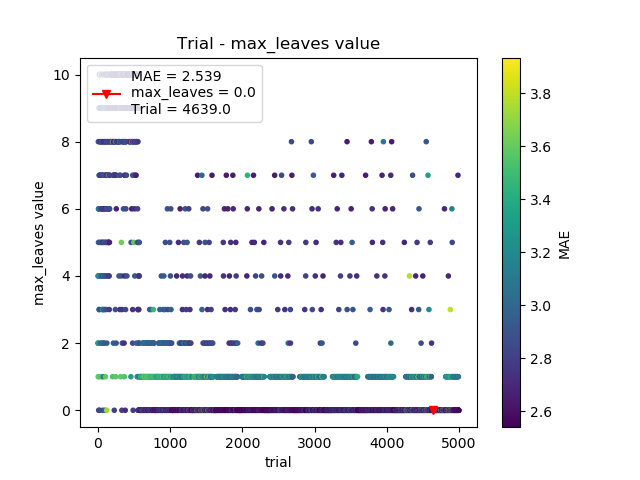

Supplement: Supplementary file 1 [file sensors-23-01162-s001.zip › sensors-2100317-supplementary/FS3.png]

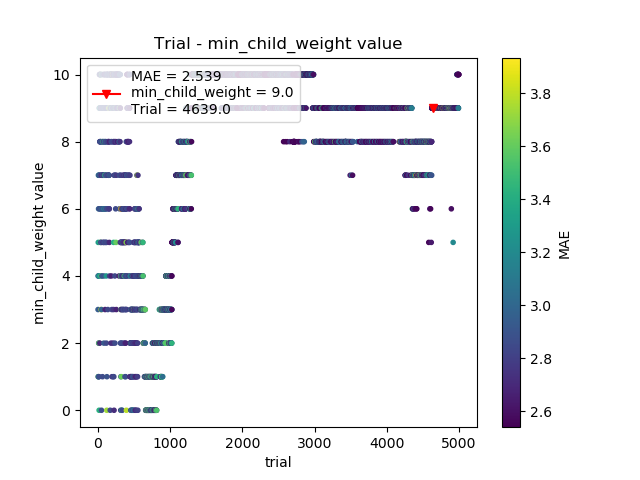

Supplement: Supplementary file 1 [file sensors-23-01162-s001.zip › sensors-2100317-supplementary/FS4.png]

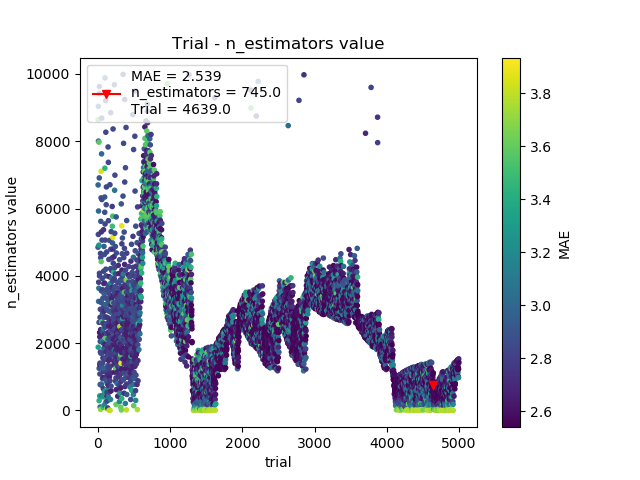

Supplement: Supplementary file 1 [file sensors-23-01162-s001.zip › sensors-2100317-supplementary/FS5.png]

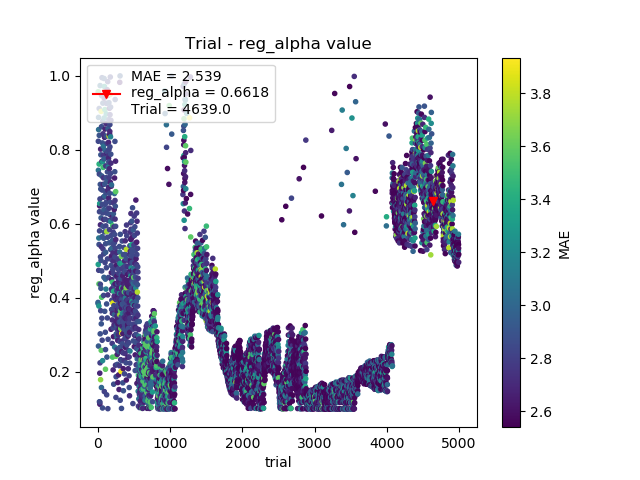

Supplement: Supplementary file 1 [file sensors-23-01162-s001.zip › sensors-2100317-supplementary/FS6.png]

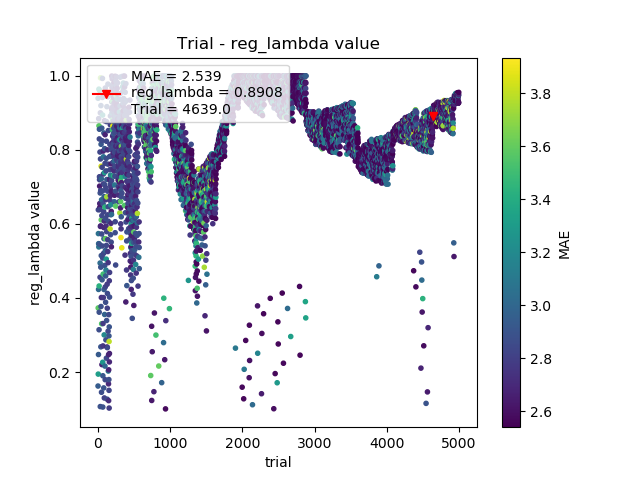

Supplement: Supplementary file 1 [file sensors-23-01162-s001.zip › sensors-2100317-supplementary/FS7.png]

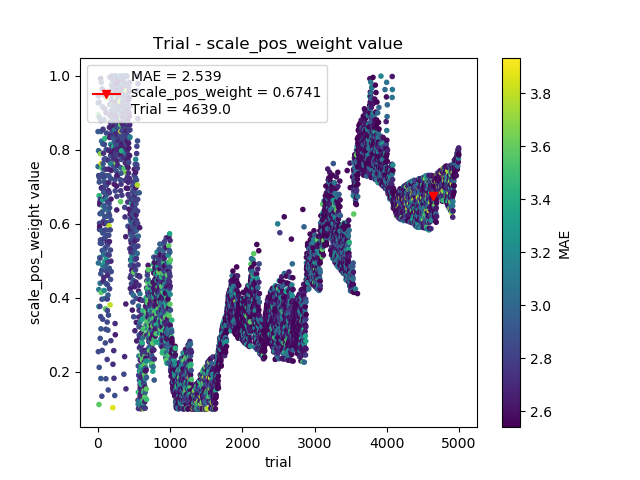

Supplement: Supplementary file 1 [file sensors-23-01162-s001.zip › sensors-2100317-supplementary/FS8.png]

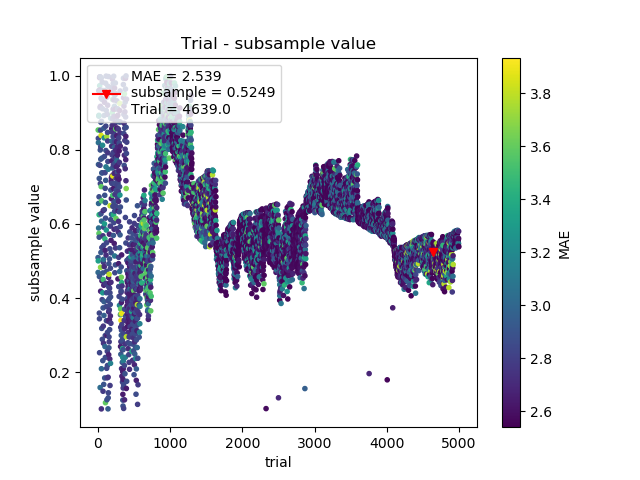

Supplement: Supplementary file 1 [file sensors-23-01162-s001.zip › sensors-2100317-supplementary/FS9.png]
